# Correlation of serum cornitine levels with bone mineral density among Indian male tobacco users

**DOI:** 10.6026/97320630018543

**Published:** 2022-06-30

**Authors:** Rashi Chauhan, Amit Kumar Singh, Manisha Singh, Richa Shree, Santosh Anand, Ashok Kumar Singh, Sankalp Verma

**Affiliations:** 1 Buddha Institute of Dental Science And Hospital, Bihar, Patna; 2Minority Dental College And Research Center, Darbhanga, Bihar, India

**Keywords:** Bone mineral density, cotinine, men, tobacco

## Abstract

It is of interest to assess levels of serum cornitine in male tobacco users and also find its correlation with bone mineral density. Assessment for serum cotinine levels was done using commercially available Enzyme Linked Immunosorbent Assay (ELISA Kit). While bone mineral density was measured using bone densitometry through ultrasound in the wrist region. Karl Pearson's coefficient was used to assess correlation between BMD values and serum cotinine (ng/ml) levels. Inter group BMD association was measured using Chi square test. The present study showed that the first 3 groups had a low BMD level compared to control group, indicative of osteopenia. BMD values were lesser for chewers from group II as compared to smokers from group I and individuals using both smoked and smokeless form of tobacco from Group III. In group III (both forms of tobacco), osteopenic individuals were more. Thus, data shows effect of tobacco usage on bone mineral density. Smokeless form of tobacco has relatively serious effects on bone density.

## Background:

Tobacco usage in any form poses a great threat to the overall human health today [1]. One-third of the world's adult population uses tobacco in one or the other form [1]. In accordance with the WHO report, approximately 5 million people worldwide passed away in 2000 due to their nicotine addiction [1-2]. About 1/3rd of tobacco users die ahead of time [1-2]. Several chronic diseases are caused by tobacco usage [5]. Besides systemic effects; tobacco also induces its ill effects on periodontium and jaw-bones [6]. Many authors have reported that consumption of tobacco results in reduced bone density [5]. The toxicity caused by nicotine, an active component of tobacco and also non-nicotine components affects BMD levels [5-6]. Nicotine has a direct effect on osteoblasts and indirectly affects calcium absorption by intestines. Osteoporosis causes greater physical disability that cancer [6, 7]. Osteoporosis, a skeletal systemic disease, renders bone fragile and brittle that makes it fracture prone. Reduced bone density is often found associated with osteoporosis which is progressive in nature. Effects of osteoporosis can be seen in any bone of the skeletal system for instance wrist, spine, pelvic, alveolar bone and tooth loss [8-14]. The correct diagnosis of osteoporosis requires thorough examination of signs and symptoms, radiographic and advanced diagnostic investigations like ultrasonography, bone scans, bone mineral density assessment. Among these diagnostic aids, ultra sonography is considered cost-effective with high accuracy with (99% sensitivity and 76% specificity [11-12]. The word serum cornitine is a synonym of serum nicotine. Cornitine is used as a biomarker or an indicator of exposure to tobacco smoke [15]. In scientific research this indicator known as cornitine is utilised as an enhancer as well as used as an antipsychotic drug. Traces of cornitine can be detected from blood post several days of tobacco use [15]. The cornitine levels in blood are directly proportionate to amount of tobacco exposure. Assessment of Cornitine level thus provides quantitative measurement of tobacco exposure and is a reliable indicator of tobacco consumption [15].Therefore it is interest to assess levels of serum cotinine in male tobacco users and also find its correlation with bone mineral density.

## Materials and methods:

### Source of data:

The study subjects were recruited from the outpatient Department of Oral Medicine and Radiology. Initially 150 subjects (40 years or above) were selected but only 120 gave their consent to enrol in the study. These 120 subjects were further divided into 4 subgroups comprising of 30 subjects in each group.

### Inclusion criteria:

The subjects having the habits of smoking, chewing tobacco, and smoking along with chewing smokeless form of tobacco were selected for the study. They were further divided into 4 subgroups:

[1] Group I comprised 30 subjects with a history of tobacco chewing at least for a period of 1 year.

[2] Group II comprised 30 subjects with a history of tobacco smoking at least for a period of 1 year.

[3] Group III comprised 30 subjects with a history of both tobacco chewing and smoking at least for a period of 1 year.

[4] Group IV comprised 30 subjects with no habits of tobacco consumption either in smoke or smokeless form.

### Exclusion criteria:

[1] Having any systemic illnesses like diabetes, hypertension etc.

[2] Taking medications that may alter bone metabolism like corticosteroids, and calcium supplements;

[3] Suffering from carcinoma and impaired renal function and

[4] Subjects unwilling to participate in the research were excluded from the study.

Assessment of bone mineral density: BMD was measured at the wrist region using ultrasound (Samsung UGEO WS80A). Participants were asked to sit relaxed and comfortably in the chair and to place their right-hand wrist on the sonography machine following which readings were recorded. Ultrasound uses sound waves to determine BMD in the wrist region.

BMD [11] is categorized on the basis of t-scores as follows (WHO)

[1] t-score -1 : Normal 

[2] t-score between - 1 to − 2.5: Osteopenic 

[3] t-score is below - 2.5: Osteoporotic 

t-score is the bone density compared with that expected in a normal healthy adult of matched age and sex. The t-score is the number of units of standard deviations (SD) that the bone density is above or below the standard. Essentially, it compares the bone density with the best possible peak bone density.

### Assessment of serum cornitine:

Following aseptic procedures about 5 ml of venous blood was drawn from the ante-cubital vein. Blood was then transferred to gel-coated tubes and transported to the laboratory for testing. Centrifugation was then performed for each sample at 3000 rpmfor 10 min. The supernatant serum obtained was then processed for quantitative estimation of serum cotinine levels. Assessment for serum cotinine levels were done for all the 4 groups using commercially available Enzyme Linked Immunosorbent Assay (ELISA Kit).

### Statistical analysis:

One-way ANOVA test (t-score) was used tocompare bone density of four study groups (Group I, Group II, Group III, and Group IV). ANOVA test was also used to compare three study groups (Group I, Group II, and Group III) with respect to log serum cotinine levels (in ng/ml).Bone mineral density was measured using bone densitometry through ultrasound in the wrist region. Karl Pearson’s coefficient was used to assess correlation between BMD values and serum cotinine (ng/ml) levels. Inter group BMD association was measured using Chi-square test.

## Results:

The present study showed that the first 3 groups had a low BMD level compared to control group, indicative of osteopenia (Table 1 - see PDF). BMD values were lesser for chewers from group II as compared to smokers from group I and individuals using both smoked and smokeless form of tobacco from Group III. In group III (both forms of tobacco), osteopenic individuals were more. Levels of serum cotinine were statistically higher in first three groups as compared to group IV or control group. (P = 0.00001).

## Discussion:

One of the serious public health issues is consumption of tobacco in any form [2]. Usage of tobacco dates back to the first-century BC. Prevalence rate is higher in Indian males than Indian females [2]. Older age group is more addicted to tobacco than the younger age groups. Tobacco usage adversely affects many organs and body systems. Heavy tobacco users are posed to highest risk of health hazards [3]. Smoke-form and smokeless form of tobacco both result in oral carcinomas, upper respiratory tract infection and lung cancers, cardiovascular diseases, preterm low birth weight babies, females may suffer from poor reproductive health, high morbidity rate [4]. Several chronic diseases are caused by tobacco usage. Besides systemic effects, tobacco also induces its ill effects on periodontium and jaw-bones [3]. Tobacco consumption is known to cause decreased bone density, which might result in osteoporosis. The effect appears to be dose-dependent and it is partially reversible [2,4]. Osteoporosis is mostly seen in females and thus most of the studies for low BMD are conducted on women subjects. Most studies of bone density have been conducted only in women.But osteoporosis due to the tobacco usage has shown deleterious effects in men [4]. Hence, the present study was conducted to evaluate the association between tobacco use and BMD in male tobacco users above 40 years of age. The word cotinine is a synonym of nicotine. Cotinine is used as an indicator ora biomarkerof exposure to tobacco smoke [16]. In scientific researched this indicator known as cotinine is utilised as an enhancer as well as used as an antipsychotic drug. Traces of cotinine can be detected from blood post several days of tobacco use [16]. The cotinine levels in blood are directly proportionate to amount of tobacco exposure. Therefore, cotinine content in the blood clearly indicates tobacco exposure [16]. Assessment of Cotinine level thus provides quantitative measurement of tobacco exposure and is a reliable indicator of tobacco consumption [16]. In this study, the use of serum cotinine levels has been evaluated as a quantitative method to assess the level of tobacco use. The absorbed dose of nicotine is best indicated by the concentration of cotinine in the blood. The relative stability of cotinine levels in blood over a time is more when compared to saliva. Cotinine levels in saliva and blood are highly correlated with saliva-to-blood ratios of 1.1:1.4.It was decided to calculate cotinine levels in the serum due to its relative stability [6]. The present study establishes the fact that cotinine levels were stable over a time and high among all the three groups when compared to control with P value being 0.00001* which was statistically significant. Thus, the serum cotinine levels can be used as reliable indicator of exposure of tobacco. According to the epidemiology; smokeless tobacco has shown its potential effect on bone metabolism. Thus, tobacco users should be evaluated thoroughly for effects on BMD and risk for osteoporosis and fracture [6]. In the present study, BMD was less among all the three groups (P = 0.2011), indicative of osteopenia. Osteopenic subjects were more in the group using both chewing form and smoke-form of tobacco. [8] But the mean density of bone was less among chewers when compared to smokers and subjects who used tobacco in both the form. This indicates that chewing form of tobacco has more ill effects on bones [8]. Spangler et al. authenticated similar results, in which he showed that nicotine or another component from smokeless tobacco adversely affects bones [6, 8]. Among the control group, 10 participants were found to be osteopenic. The probable etiology for the subjects having ostepenia without having tobacco habit could be because of nutritional insufficiency of calcium, vitamin D, or passive smoking [8]. Hence, it is important to identify individuals with low BMD by screening for osteoporosis, particularly among patients with tobacco habit and risk of osteoporosis.

## Conclusion:

The ill effects of nicotine and tobacco exposure by serum cotinine are described using ELISA to diagnose low BMD. This method is easily approachable, cost-effective and feasible for identifying osteopenia and osteoporotic conditions. Delay in these measures might have serious complications when bones become fragile and fracture prone. Early detection of serum cornitine can be used for surveys at bigger scale encompassing a whole community to identify the extent of the issue in question. Data shows the effect of tobacco usage on bone mineral density. Smokeless form of tobacco has relatively serious effects on bone density.
